# Are emotions contagious? A conceptual review of studies in language education

**DOI:** 10.3389/fpsyg.2022.1048105

**Published:** 2022-10-21

**Authors:** Yu Kong

**Affiliations:** Department of Foreign Languages, Shenyang Normal University, Shenyang, China

**Keywords:** emotions, language education, second language acquisition (SLA), English as a Foreign Language (EFL), emotion contagion

## Abstract

Considering the great role those affective variables play in language learning, it seems wise to hypothesize whether teacher and learner emotions are contagious in the language classroom or not. The existing literature on emotion contagion in other fields of study accepts this hypothesis and reveals significant points about the verbal and non-verbal cues for emotion contagion in class. The present paper introduces and defines the topic and goes on with summarizing the procedure of transmitting the emotion. Then, it reviews the existing research on emotion contagion in different areas and creates a link between them and the L2 studies. It draws attention to the dearth of research on emotion contagion in the second language acquisition (SLA) domain and emphasizes the need for innovative dynamic approaches to research to explore emotion contagion in the ecology of the English as a Foreign Language (EFL) classroom. It also reviews the limited body of research in this regard in the SLA domain and shows how the significant findings can be useful for L2 educators. The findings of these studies show that more relevant studies in the future can be hoped to provide insightful remarks about how different emotions are transmitted between the teacher and students or among students in L2 classes. These studies are supposed to adopt a dynamic approach as well as a longitudinal data collection and analysis procedure. These can have implications for teacher education programs in the English language teaching (ELT) domain.

## Introduction

Emotion contagion is an event marked by the observed behavior of an individual resulting in the reflexive manifestation of the same behavior by some other individuals (Nickerson, 2021). It appears to be a general phenomenon in human communities. Yet, we show here how relevant it is to the language (L2) learning domain, how it can happen and why it is necessary to approach it from a dynamic view in L2 classrooms. The paper begins with the overall definition and theoretical background and moves on to review works of research on emotion contagion in the ecology of L2 classrooms. The implications for L2 teachers and learners will be provided.

When the observed behavior of an individual is copied by others and the same emotion rises, the phenomenon is known as emotion contagion (Panksepp and Lahvis, 2011). Emotional contagion is approached as the process through which individuals’ emotions are affecting or being affected by each other, probably unconsciously or maybe partly consciously (Hatfield et al., 1993). Some emotions may be transferred through verbal accounts, facial expressions, gestures, postures, and other similar behaviors from an individual to those observing him/her (Hatfield et al., 1994). Recognizing emotions from facial expressions is universally shared among different cultures (Brown, 2004), as facial expressions can convey emotions showing approval, expectations, or more intense feelings (Nickerson, 2021).

The roots of emotion contagion are found in a body of research that showed individuals observing a facial expression produce an emotional response that copies the emotions shown by the individual who first revealed that facial expression (Lundqvist and Dimberg, 1995; Hess and Blairy, 2001). Researchers such as Barger and Grandey (2006) reckon that this act of copying is done to empathize with others or get linked to them. Hatfield et al. (1993) claim that since 1759, economic philosophers including Adam Smith realized that individuals tend to view themselves in each other’s shoes and show copying behaviors. The Emotional Contagion Hypothesis was first put forth by Hatfield et al. (1993) to define emotion contagion as the capability of automatically copying and synchronizing vocalizations, expressions, postures, and movements with the same act and emotions expressed by others, consequently to emotionally converge to them.

The early sources show there are several ways to receive emotions. For example, according to Adam Smith, these sources include a conscious application of logic, analysis, and conceptualization. According to Giuliana and Carvalho (2016), some individuals are more likely to convey or receive emotions than others. Verbeke (1997) classified these individuals in two groups, including the powerful emotion transmitters, and the powerful emotion receivers. Emotion contagion can happen through different channels for example aural and visual channels, together or each on its own. For instance, a commonly cited reason for listening to music is that it can elicit intense emotions (Juslin and Laukka, 2004). Yet, Hatfield et al. (1993) suggested several propositions as the reasons for emotional contagion and contended that the precursors of emotional contagion are more ubiquitous, subtle, and automatic than the former theorists thought. The two main causal mechanisms suggested by Hatfield were feedback and mimicry.

## The procedure of emotion contagion

According to Lundqvist and Dimberg (1995), the first step of emotional contagion is that an individual gives an automatic and unconscious response to the expressions that are seen around and known to be an impetuous imitation of other people’s facial expression. This automatic mimicry is, thus, unconscious copying of movements and speech, facial expressions, gestures, and eye contact (Chartrand and Van Baaren, 2009). Automatic mimicry is capable of recognizing emotions (e.g., Stel and van Knippenberg, 2008; Neal and Chartrand, 2011; Wood et al., 2016) and sharing feelings (e.g., Decety and Lamm, 2006; Schuler et al., 2016). This automatic mimicry is inborn as witnessed even in the newborn (Field et al., 1982; Haviland and Lelwica, 1987). The basal motor imitation (Condon and Sander, 1974; Bavelas et al., 1986; Bernieri et al., 1988) can result in the creation of an internal cue (Cacioppo et al., 1988; Strack et al., 1988; Dezecache et al., 2015; Wang and Guan, 2020) and experiencing and sympathizing with other individuals’ emotion (Allport, 1961; Barsade et al., 2018). As explained by Wood et al. (2016), individuals experience the imitation of an emotional expression when they see it in other individuals.

The next step is that when individuals copy a behavior, they begin to wholeheartedly feel the same emotion (Laird and Bresler, 1992; Hatfield et al., 1994). When individuals imitate their partners’ emotional expressions unconsciously, they begin to sense some manifestations of the same emotions too (Prochazkova and Kret, 2017). Following another individual’s behavior can cause emotion contagion (Cacioppo et al., 2000), which implies giving emotional responses to the emotions expressed (Hatfield et al., 1994), yet that does not merely involve giving a response to facial expressions. It is argued that mimicry is the main mechanism of action underlying emotion contagion (Hatfield et al., 1994), so we cannot see it necessarily as an equivalent for emotion contagion. For instance, if several learners in a class smile at their teacher or the other students, it does not necessarily end in a positive emotion transmitted (Elahi Shirvan and Talezadeh, 2018). Therefore, emotion contagion can be concluded to be a higher-order construct not necessarily associated with a particular form of mimicry (Prochazkova and Kret, 2017).

## Research on emotion contagion and implications for the language domain

Given the advent of complex dynamic systems theory in the field of second language acquisition (SLA), emotions are no longer seen as traits but they are supposed to be viewed as states. This dynamic perspective can contribute to the understanding of the contagious nature of emotions during the process of L2 learning because it enables to trace the moment-to-moment emergence of the emotion contagion. Emotion contagion research has revealed that exposure to emotional expressions can induce a change in the onlooker’s emotional condition. These emotional conditions arise unconsciously and are initiated by cues in the environment that distinctively affect people’s mood (Berntsen, 2007). McHugo et al. (1985) reported that viewing the portrayal of a person who was smiling led to a change in the face expression of the onlooker. This is known as the primary emotion contagion marked by a tendency to automatically copy and mimic vocalizations, facial expressions, movements, and postures with those of another individual and, as a result, to emotionally converge to it (Hatfield et al., 1994).

Emotion contagion is explored in several areas such as positive and negative information assessment (Hamilton and Zanna, 1972; Kanouse and Hanson, 1972; Crandall, 1975) and passenger encouragement by flight attendants to create a desirable mood (Hochschild, 1983). In social neuroscience, emotions are also perceived to be capable of eliciting autonomic and somatic responses, as well as the same neural representation of the emotional condition that an individual is experiencing (e.g., Gallese and Goldman, 1998; Goldman and Sripada, 2005; Keysers and Gazzola, 2010; Anders et al., 2011). Admitting that social and complex stimulations can cause emotion contagion (Hatfield et al., 1994), we should be mindful of the emotional clues (e.g., the muscles of face, eye contact, size of the pupils, body postures, and blushing) (Prochazkova and Kret, 2017) in emerging and complex situations, including the foreign language learning context.

There is currently a dearth of research on the underlying procedural mechanisms of emotion contagion in the foreign language learning context. The language studies domain has also faced a recent domain-specific turn from negative to positive psychology; thus, there is a further need to explore the positive emotion contagion. How emotions may be recognized is seemingly more evident to others than it really is (Gilovich et al., 1998). Others notice emotions through non-verbal clues including facial expression gestures, vocalics and body language (Mehrabian and Epstein, 1972). Nevertheless, sometimes these emotions are recognized incorrectly (Gregersen et al., 2017; Elahi Shirvan and Talezadeh, 2018; Fan and Wang, 2022). Therefore, it is essential to look for insightful information about the underlying mechanisms of emotion contagion in the foreign language learning context, now that we acknowledge the truth of this event.

Conducting research on emotion contagion in the foreign language learning context needs an exhaustive consideration of non-verbal signals. A variety of codes make up non-verbal communication such as kinesics including body language, posture, and gesture (Wallbott and Scherer, 1986; Chartrand and Bargh, 1999), emotional energy (the intensity of emotion expression) such as pitch range, pitch level, tempo and loudness (Scherer, 1981), vocal tones (Hatfield et al., 1994; Hietanen et al., 1998; Neumann and Strack, 2000), facial expressions including smiling and eye contact (Wallbott and Scherer, 1986) and such vocalics as speech patterns (e.g., Ekman et al., 1976). These non-verbal signals, in negative psychology, have already pointed to the existence of anxiety (Gregersen et al., 2017). Due to the unclear boundary between imitation and emotion contagion in the existing literature, researchers should employ dynamic approaches for exploring emotion contagion in L2 classrooms.

In the SLA research, the dynamic shift (Dörnyei and Ryan, 2015), in the light of the complexity and dynamic systems theory (CDST) is a change from linear and isolated perspectives to a dynamic and non-linear approach to affective constructs (Ellis and Larsen-Freeman, 2006; de Bot et al., 2007; Larsen-Freeman and Cameron, 2008). MacIntyre and Legatto (2011) contended that exploring the notion of moments of variation in individuals can be employed in the applied linguistics domain. To investigate the temporal changes in emotion development in class and capture the moment-by moment variations, several innovative research methods have been introduced which are compatible with the dynamic turn in applied linguistics. More specifically, the research on emotion contagion in the field of SLA has been intertwined with the application of the innovative methodological approaches in this field. Some are quantitative and some qualitative, and are being used within the past few years of affective variables in L2 studies. Examples of the former are the latent growth factor modeling (LGCM), and panel designs. Examples of the latter are process tracing, the idiodynamic method, concept mapping and qualitative comparative analysis. These innovative dynamic methods of research seem to be appropriate for investigating emotion contagion in an L2 class, as they are all longitudinal in type, and can trace the momentary changes in emotions and how they develop or co-develop among L2 teachers, language learners and both. We will review the existing related literature in the SLA research.

## L2 studies of emotion contagion

In education, the growth of humanistic psychology has been accompanied by more attention to and interest in affective variables (Bao and Liu, 2021). Investigating emotions in SLA studies goes back to the late twentieth century, when positive psychology was still absent, and attempts were made more to decrease the negative affective variables that could hinder language learning such as L2 anxiety. From the late 1990s, the rate of investigating affective variables has increased rapidly. In the twenty first century, SLA entered a new phase of emotion investigation characterized by a concern for both negative and positive emotions (Dewaele and Li, 2020). Positive psychology was introduced to SLA research in 2010, and newer emotions such as foreign language boredom (Derakhshan et al., 2021; Pawlak et al., 2021), foreign language enjoyment (MacIntyre and Mercer, 2014; MacIntyre and Gregersen, 2022) and loving pedagogy (Wang et al., 2022) were also introduced. Concerning emotion contagion, the body of research in SLA studies is still limited, and this field yet waits for more productive studies.

Talebzadeh et al. (2020) aimed to identify and trace the non-verbal signals in the development of foreign language enjoyment concerning the potential underlying mechanisms accounting for enjoyment contagion in the ecology of an L2 class. They employed an idiodynamic method in five dyadic rounds of student and teacher interactions. The idiodynamic approach had been developed by MacIntyre and Legatto (2011) to investigate the moment-to-moment changes of affective variables. This research method is suggested to researchers who hope to trace the momentary changes in an emotion longitudinally. The results of the study by Talebzadeh et al. (2020) showed that automatic mimicry is the main causal mechanism of enjoyment contagion in the target interactions. It is formed by using gestures, facial expressions, and postures including vocalic expressions, laughter, nodding, smiling, and leaning forward. Nevertheless, concerning the contribution to the dynamics of enjoyment contagion, the mimicry does not necessarily translate into enjoyment.

Another emotion that was investigated in terms of emotion contagion between the teacher and students in an L2 classroom is happiness. Moskowitz and Dewaele (2019) investigated the association between perceptions of L2 teacher happiness and L2 learners’ attitude. These researchers first drew attention to the lack of research on how student perception of teacher happiness affect language learners’ emotions and attitudes. Then, they used an online survey to collect data from 129 adult English language learners from different parts of the world, who had participated in formal English intermediate to advanced level of proficiency classes. The students were asked to share their attitudes toward the different features of their teachers’ happiness, and about their own perception of and willingness to learn English. Statistical procedures showed that language learners’ attitude to teacher happiness was positively and significantly correlated with language learners’ overall motivation and attitude, as well as language learners’ perception of the teacher. This is understood as a manifestation of the mechanism of positive emotional contagion between students and teachers.

As evident, there are still many L2 teacher- and learner-related emotions that can and need to be explored in terms of contagion in an L2 class. These can include teachers’ and learners’ boredom and anxiety. Though the existing literature on emotion contagion in L2 classrooms is still limited, the aforementioned studies proved strong associations between the emotions of L2 learners and teachers. Thus, as suggested by Moskowitz and Dewaele (2019), it can be argued that teachers should and could actively try to turn teaching into a positive experience not just for their own sake but also for their language learners. In practice, we should admit that this is occasionally easier to say than to do. The reason is that in practice, many uncontrollable variables can usually be involved, including professional pressure, personal issues or institutional culture. Still, these potential limitations cannot discourage the continued line of research on how emotions are transmitted among language learners or between the teacher and the language learners in class. Investigating language learners’ perceptions of language teachers’ emotions is still relatively new. Yet, conceiving of an emotional relationship between teachers and learners and the significant effect that the student-teacher association has on the language learning experience, investigating how language learners understand their teachers’ feelings and mood is a promising topic of research that awaits further development in near future. The language teacher’s and learners’ emotions are not only important on their own, but they lie at the heart of the learning experience and, thus, need to be examined more deeply, as emphasized by some researchers (Mercer and Kostoulas, 2018; Wang et al., 2021).

## Conclusion

Recent years have witnessed a growing interest in investigating affective constructs in L2 studies. As already described, the initial interest in negative emotions and trying to lower them was followed by the introduction of positive psychology and a new line of research on both positive and negative emotions in SLA research. Also, influenced by the dynamic turn of the century, the introduction of new research methods (e.g., idiodynamic approach, process tracing, interaction analysis, etc.) made it possible to conduct longitudinal studies on momentary changes in the developmental nature of the emotions that the teacher and language learners experienced in an L2 class. An interesting fact is that the ecology of a foreign language classroom hots many interactions between the teacher and language learners, and also among the language learners, which paves the way for the transmission of emotions. The kind of information exchange in these interactions is by no means limited to the verbal transmission of information. Admittedly, an emotional side is also saliently involved. In other words, during the teacher and student interactions, not only is the information transferred but also the emotions are conveyed.

Similarly, student-student interactions can accommodate a varying intensity of different emotions transferred among students in pair or group work tasks and activities. Certainly, the teacher can also get involved in these interactions too by, for example, monitoring students’ pair or group work and providing occasional feedback (Derakhshan et al., 2022). The feedback does not have to be only verbal. Non-verbal feedback can be as effective in transmitting emotions, especially as facial expressions, eye contact, different gestures and postures of body (see Bayat et al., 2020). Thus, the emotion contagion topic of research in an L2 classroom can address many different emotions (not yet explored in the literature) and many nuances of changes in verbal and non-verbal cues. The existing literature on emotion contagion in L2 classes is still limited. More specifically, the literature of emotion contagion in the field of SLA has been mainly limited to positive emotions. L2-related emotions such as foreign language enjoyment or teacher happiness showed to have been contagious in L2 classes. However, this does not mean that this contagion cannot take place in the experience of negative emotions such as anxiety and boredom. Thus, other emotions are worth investigating as well.

## Pedagogical implications

Emotion contagion has proved to be a promising topic of research in L2 studies. From a positive psychology perspective, experiencing positive emotions can enhance L2 leaners’ mental capacity and broaden their attentional scope. Thus, language teachers’ emotional support in the environment of L2 classes enables their learners to take full advantage of their cognitive repertoire in the process of language learning. Though the existing literature in the SLA domain is still limited, the research findings can raise teachers’ awareness of what they can do to increase positive emotions in teacher-student interactions with their L2 learners in class (Xie and Derakhshan, 2021). In other words, the contagious nature of emotions in the field of SLA highlights the salient role of emotional support on the part of language teachers and peers in the environment of L2 classes. In this emotional support, they can raise their learners’ awareness of the contagious nature of emotions and how they can help each other by transferring their positive emotions to each other in their classroom conversations. Teachers are also recommended to adequately attend to the different forms of cues they may apply in within-class interactions including smiling, laughing, eye contact, other facial expressions, body movements and overall gestures. The chances for emotion contagion should be discussed actively in teacher training courses. Pre- and in-service language teachers should be made aware of the significance of showing positive emotions in class and how they can convey these positive feelings to students in the classroom interactive events. Instances are the feedback provision, Q&As, warm-up activities and so on. Training courses are, thus, expected to represent how to create positive emotion contagion and how to control it and organize its dynamics during the classroom interactions for the pre-service and in-service teachers. L2 teachers need guidance on how to construct a positive emotional climate of language learning for the students. Besides, this awareness can be conveyed to students as well to make them involved in the development of positive emotion contagion in interactions with their classmates or their teacher.

L2 teachers and learners both need to become aware that any mimicry seen among the interlocutors within an interaction may not be a sign of the contagion of an emotion. A lacking awareness can cause misunderstandings about the teacher’s or students’ actions or a lack of correspondence with their expectations. Still, there is a significant need for more research to reveal facts about the underlying mechanisms of emotion contagion in L2 classes, before we can suggest how mimicry can be translated into contagion.

## Suggestions for further research

In fact, the dynamic phase of exploring L2 affective variables has only begun since a few years ago. Just a handful of affective variables have been studied so far longitudinally and dynamically. Many others are left unexplored yet through many robust dynamic methods of research. Hope, social aloofness, loneliness, learner immunity and compassion are among the unexplored L2 affective variables in the dynamic turn of SLA research.

Exploring the contagion of enjoyment and happiness (addressed in the existing literature) can also be further developed to and linked with the other aspects of the classroom discourse such as teacher-originated feedback for language learners’ answers and comments and also the teacher’s negative/corrective feedback. It is suggested that innovative research methods be employed to explore emotion contagion in L2 studies. Conventional methods of analysis with their one-shot designs fail to capture the true developmental nature of L2 emotions leave be the emotion transmission. Thus, future researchers are advised to use innovative dynamic research methods that are longitudinal in type so that they can represent the momentary variation in the emotions. Examples were already suggested in this paper for both qualitative and quantitative research. Talebzadeh et al. (2020), for example, suggested that the further investigation of enjoyment contagion be conducted using other analytical approaches such as the conversation analysis.

As already raised, investigating L2 learners’ attitudes to their teachers is still relatively new. However, due to the potential emotional association between L2 teacher and learners and the significant effect of teacher-student interactions on the language learning process, delving into how L2 learners perceive their teachers can be a promising area of research that deserves further exploration too.

There are hopes that the few works of qualitative and quantitative dynamic research on L2 emotion contagion reviewed here pave the way for more insightful and in-depth studies in near future. The dynamic line of inquiry in SLA holds promises for delineating a more realistic picture of how affective traits emerge out of the complexity of classroom learning, and how and why they show different patterns of change and transmission within and between individual language learners and their teacher in class. Also, gestures can provide a better understanding of the process and mechanisms of emotion contagion in L2 learning. To explore the role of gestures in the emergence of emotional contagion, a multimodal perspective is suggested. This multimodal perspective enables researchers to explore what gestural mechanisms can contribute to the emergence of this contagion.

## Author contributions

The author confirms being the sole contributor of this work and has approved it for publication.
